# The Survival Benefits of Surgical Resection and Adjuvant Therapy for Patients With Brainstem Glioma 

**DOI:** 10.3389/fonc.2021.566972

**Published:** 2021-03-25

**Authors:** Zhuoyi Liu, Songshan Feng, Jing Li, Hui Cao, Jun Huang, Fan Fan, Li Cheng, Zhixiong Liu, Quan Cheng

**Affiliations:** ^1^ Department of Anesthesiology, Xiangya Hospital, Center South University, Changsha, China; ^2^ Department of Neurosurgery, Xiangya Hospital, Center South University, Changsha, China; ^3^ National Clinical Research Center for Geriatric Disorders, Xiangya Hospital, Central South University, Changsha, China; ^4^ Xiangya Cancer Center, Xiangya Hospital, Central South University, Changsha, China; ^5^ Key Laboratory of Molecular Radiation Oncology of Hunan Province, Changsha, China; ^6^ Department of Rehabilitation, Second Xiangya Hospital, Central South University, Changsha, China; ^7^ Department of Psychiatry, The Second People's Hospital of Hunan Province, The Hospital of Hunan University of Chinese Medicine, Changsha, China; ^8^ Center for Medical Genetics & Hunan Provincial Key Laboratory of Medical Genetics, School of Life Sciences, Central South University, Changsha, China; ^9^ Department of Emergency, Fengyang County Hospital of Traditional Chinese Medicine, Fengyang, China; ^10^ Department of Clinical Pharmacology, Xiangya Hospital, Central South University, Changsha, China

**Keywords:** brainstem glioma, SEER, adjuvant therapy, gross total resection, cancer specific survival

## Abstract

**Purpose:**

The role of surgical resection in the treatment of brainstem glioma (BSG) is poorly understood. For pediatric low-grade (LGBSG) group, several monocentric small-scale retrospective studies reported contradictory conclusions. And there was no clinical study focused on surgical resection for adult or pediatric high-grade (HG) patient groups. This study aims to illustrate whether surgical resection and adjuvant therapy provide survival benefits for patients with histologically confirmed BSG.

**Patients and Methods:**

This retrospective cohort study included 529 patients with histologically confirmed BSG in Surveillance Epidemiology and End Results (SEER) database from 2006-2015. Patients were divided into four groups by age and World Health Organization (WHO) grade. Kaplan-Meier curves of CSS were plotted by different treatment options to compare the survival probability. Univariate and multivariable analyses were then conducted to determine the prognosis effects of surgical resection and adjuvant therapy on cancer specific survival (CSS). All analyses were done in four different groups separately.

**Results:**

The final sample included 529 patients. The entire study population was divided into groups of pediatric LG (n=236, 44.6%), pediatric HG (n=37, 7.0%), adult LG (n=204, 38.6%) and adult HG (n=52, 9.8%). 52.7% (n=144) of pediatric patients had pilocytic astrocytoma and 45.3% (n=116) of adult patients had ependymoma. Pediatric LGBSG group had the highest gross total resection (GTR) rate (61.4%) and 5-year CSS rate (88.6%). Kaplan-Meier curves of pediatric LGBSG group revealed that patients treated with GTR had significantly better survival probability (P=0.033). Multivariable analysis identified GTR as independently significant predictor for prolonged CSS in pediatric LGBSG group (HR0.29, 95%CI 0.11-0.78, P=0.015); Surgical resection showed no relation to CSS in other patient groups. Kaplan-Meier curves of adult HGBSG group showed that patients treated with both RT and CT in adult HGBSG group had the best survival probability (P=0.02). However, multivariable analysis showed the combination of radiotherapy (RT) and chemotherapy (CT) was not significantly related to better CSS in adult HGBSG group (HR0.35, 95%CI 0.11-1.09, P=0.070). Adjuvant therapy didn’t associate with better CSS in other patient groups.

**Conclusion:**

Pediatric LGBSG group had the highest GTR rate and the most favorable clinical outcome. GTR can provide significant survival benefits for pediatric LGBSG group.

## Introduction

Brainstem glioma (BSG) constitutes 10.9% of pediatric brain tumor and about 2.5% of adult brain tumor according to the most recent Central Brain Tumor Registry of the United States (CBTRUS) report (1). As there is no standard definition of glioma in World Health Organization (WHO) classification, we used the definition of glioma from CBTRUS, which includes astrocytoma, oligodendroglioma, ependymoma, oligoastrocytoma (mixed glioma), malignant glioma, not otherwise specified, and a few rare histologic types. Choux et al. classified BSG into types of diffuse, intrinsic focal, extrinsic focal and cervicomedullary based on imaging characteristics and surgical experience (2). Pediatric patients with diffuse brainstem pontine glioma (DIPG) were reported to have dismal prognosis and primarily treated with radiotherapy (RT), which was highly infiltrative and less amenable to surgery (3, 4). Recent studies reported that pediatric DIPG was associated with H3K27M mutation, which was strongly suggested to be a developmental mutation confined to brainstem development (5, 6). BSG without such typical appearances and mutations requires careful assessment as their age group and tumor grading may be highly informative about their amenability to surgical management and need for adjuvant therapy. Surgical resection is an important initial treatment for glioma, and gross total resection (GTR) is related to better survival in both WHO low grade (LG, grade I and II) and high grade (HG, grade III and IV) gliomas (7, 8). However, the role of surgical resection for BSG is not fully understood. Before the era of MRI, BSG was considered as malignant and unresectable. With the advancements of neuroradiological, anesthesiological and neurosurgical techniques (9–11), attempts of surgical resection have been made in both pediatric and adult patients with BSG (12–14), especially in pediatric patients with LGBSG (15–17). Several cohorts reported that pediatric patients with LGBSG treated with surgical resection had favorable clinical outcomes, but most of them were monocentric small-scale studies with selective patients (18–20). More recently, a prospective cohort with 116 pediatric patients with LGBSG reported that higher extent of surgery was related to better progression free survival but not to overall survival (15). As a result of relatively low prevalence and percentage of surgical resection, clinical study focused on surgery still lacks in pediatric patients with high grade BSG (HGBSG) and adult patients with BSG. There were two clinical studies including more than 100 patients (101 and 104) for adult patients with BSG. However, the percentage of surgical resection was too low to determine its effects on survival (10.6% and 13.9%) (21, 22). In summary, for pediatric LGBSG group, the benefits of radical resection on survival are still controversial. For other patient groups, the role of surgical resection is poorly understood.

Our previous study developed an effective nomogram to predict the clinical outcome of patients with LGBSG. Nevertheless, the cohort it analyzed is a mix of pediatric and adult patients (23). BSG patients with different age and WHO grade had different clinical characteristics, prognosis and treatment options. Thus, horizontal comparison between these subgroups of BSG is of great necessity. This study took advantage of Surveillance Epidemiology and End Results (SEER) database to include 529 patients with histologically diagnosed BSG. Patients were divided into groups of pediatric LGBSG, pediatric HGBSG, adult LGBSG and adult HGBSG. This work highlighted the differences in clinical outcome and treatment options between different patient groups. Also, we analyzed the effects of surgical resection and adjuvant therapy on caner specific survival (CSS) in different patient groups separately.

## Method

### Study Population

The initial sample included patients with primary diagnosed central nervous system tumor (2004–2015) recorded in SEER database. SEER database is the largest cancer data set maintained by the National Cancer Institute, which collects data from 19 national geographic states and covers 34.6% of America population. It has high quality data with standardized collection practice and de-identified information. Firstly, patients with tumor located in brainstem (code C71.7) and positive histology were included. Secondly, the patients with pathological diagnosis of glioma were selected. Because there is no standard definition of glioma in WHO classification, we used the definition from the most recent CBTRUS, which includes ICD-O-3 histology codes 9380–9384, and 9391–9460 in accordance with the recode rules for 2007 WHO Classification of CNS tumors. Thirdly, patients without important information including race, marital, size, extension, metastasis, surgery status, and CSS status were excluded. Lastly, the patients treated with no surgery (code 00) were excluded. The final study population strictly included patients treated with biopsy (code 20), subtotal resection (STR, code 21), or gross total resection (GTR, codes 30, 40, and 55) (**Supplement Figure 1**).

### Covariates Included

Demographic data obtained for analysis were: age of diagnosis (≤17 years old were assigned to pediatric group, >17 years old were assigned to adult group), sex, race (white, black and other) and marital status (yes, no). The tumor characteristics data included for analysis were: Tumor size (size ≤4.8 cm, size > 4.8 cm, the best cut-off value was defined according to X-tile software), WHO grade (WHO grade I and II were defined as low grade, WHO grade III and IV were defined as high grade), metastasis (yes, no), tumor extension (brainstem, cerebellum, ventricular and other). Regarding treatment options, extent of surgery [biopsy, subtotal resection (STR), and GTR] and adjuvant therapies [RT, chemotherapy (CT), both, and none/unknown] were analyzed.

### Statistical Analyses

The frequency distribution between patients treated with Biopsy, STR and GTR was determined by chi squared test. Patients were divided into groups of pediatric LGBSG, pediatric HGBSG, adult LGBSG and adult HGBSG by age group and WHO grade. Kaplan-Meier curves were plotted by GTR and non-GTR in four different patient groups. Kaplan-Meier curves were also plotted by different adjuvant therapies in four different patient groups. The differences of survival probability were compared by log-rank test. Both univariate and multivariable analyses of all covariates were done in four different patient groups to estimate the prognosis effects of surgical resection and adjuvant therapy on CSS. Hazard ratios (HRs) and corresponding 95% confidence intervals (CIs) were calculated. P<0.05 was defined as statistically significant.

## Result

529 patients were included in the study population. The demographic information, tumor characteristics and treatment options were reported in **Table 1**. The mean age of the entire cohort is 24.97 (0-85) years old and the 5-year CSS rate is 74.1%. Among them, 127 (24.0%) patients were treated with biopsy only, 109 (20.6%) patients received STR and 293 (55.4%) patients underwent GTR. At the time of data collection, the CSS rates of biopsy, STR and GTR group were 59.1%, 67.0% and 76.8% respectively (P<0.001). The overall survival rate of GTR group was also the highest (P<0.001). The GTR rate was significantly higher in patients with LGBSG (262/440, 59.5%) than patients with HGBSG (31/89, 35%, P<0.001). The GTR rate is slightly different for tumor confined within brainstem (114/226, 50.4%), tumor extended to cerebellum (26/59, 44%), ventricular system (97/152, 63.8%) and other (56/92, 61%, P=0.059).

**Table 1:** Patient demographics, tumor characteristics and treatment options of 529 patients with histologically confirmed BSG.

Characteristics	ALL N (%)	Biopsy N (%)	STR N (%)	GTR N (%)	P-Value
Population Size	529 (100)	127 (24.0)	109 (20.6)	293 (55.4)	
Age group					0.27
Pediatric	273(51.6)	58 (11.0)	56(10.6)	159 (30.1)	
Adult	256 (48.4)	69 (13.0)	53 (10.0)	134 (25.3)	
Sex					0.5152
Female	252 (47.6)	64(12.1)	55 (10.4)	133 (25.1)	
Male	277 (52.4)	63 (11.9)	54 (10.2)	160 (30.3)	
Race					0.509
Other	36 (6.8)	10 (1.9)	9 (1.7)	17 (3.2)	
Black	55 (10.4)	17 (3.2)	12 (2.3)	26 (4.9)	
White	438 (82.8)	100 (18.9)	88 (16.6)	250 (47.3)	
Marital status					0.425
Unmarried	362 (68.4)	81 (15.2)	77 (14.6)	204 (38.6)	
Married	167 (31.6)	46 (8.8)	32 (6.0)	89 (16.8)	
WHO grade					<0.001^†^
Low grade	440 (83.2)	95 (18.0)	83 (15.7)	262 (49.5)	
High grade	89 (16.8)	32 (6.0)	26 (4.9)	31 (5.9)	
Size					0.677
<4.8cm	417 (78.8)	103 (19.5)	87 (16.4)	227 (42.9)	
≥4.8cm	112 (21.2)	24 (4.5)	22 (4.2)	66 (12.5)	
Extension					0.059
Brainstem	226 (42.7)	62 (11.7)	50 (9.4)	114 (21.6)	
Cerebellum	59 (11.2)	18 (3.4)	15 (2.9)	26 (4.9)	
Ventricular	152 (28.7)	31 (5.9)	24(4.5)	97(18.3)	
Other	92 (17.4)	16 (3.0)	20 (3.8)	56 (10.6)	
Metastasis					0.943
No	517 (97.7)	124 (23.4)	107 (20.2)	286 (54.1)	
Yes	12 (2.3)	3 (0.6)	2 (0.4)	7 (1.3)	
Adjuvant therapy					<0.001^†^
None/unknown	237 (44.8)	54 (10.2)	41 (7.8)	142 (26.8)	
Both	105 (19.8)	34 (6.4)	33 (6.2)	38 (7.2)	
Radiotherapy	138 (26.1)	31 (5.9)	21 (4.0)	86 (16.2)	
Chemotherapy	49 (9.3)	8 (1.5)	14 (2.6)	27 (5.2)	
Vital status					<0.001^†^
Alive	373 (70.5)	75 (14.2)	73 (13.8)	225 (42.5)	
Dead	156 (29.5)	52 (9.8)	36 (6.8)	68 (12.9)	
Cancer specific vitals					<0.001^†^
Alive	397 (75.0)	81 (15.3)	77 (14.6)	239 (45.2)	
Dead	132 (25.0)	46 (8.7)	32 (6.0)	54 (10.2)	

^†^P < 0.05, statistically significant.

**Figure 1:**
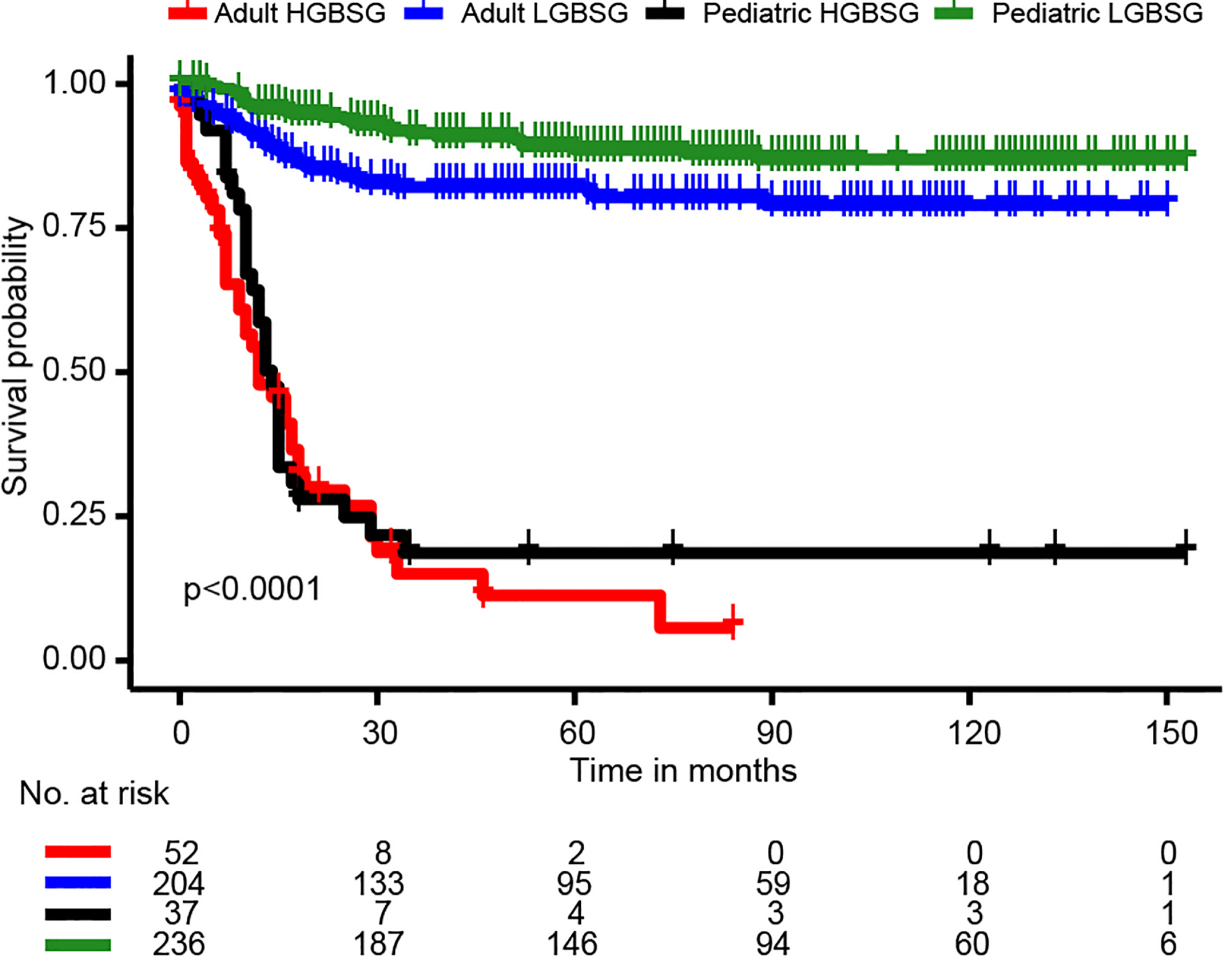
Kaplan-Meier curves for patients in different patient groups.

The entire study population was divided into groups of pediatric LGBSG (n=236, 44.6%), pediatric HGBSG (n=37, 7.0%), adult LGBSG (n=204, 38.6%) and adult HGBSG (n=52, 9.8%). The Kaplan-Meier curve by four patient groups showed that pediatric LGBSG group had the most favorable clinical outcome (P<0.001) (**Figure 1**). The 5-year CSS rates of pediatric LGBSG, pediatric HGBSG, adult LGBSG and adult HGBSG group were 88.6%, 18.6%, 82.1% and 11.3% respectively. Concerning treatment options, adult HGBSG group had the highest percentage of biopsy (21/42, 40.4%) and pediatric HGBSG group had the highest percentage of STR (12/37, 32.4%). The GTR rate was higher in pediatric LGBSG group (145/236, 61.4%) and adult LGBSG group (117/204, 57.4%) than pediatric HGBSG (14/37, 37.8%) and adult HGBSG (17/52, 32.7%) group (P<0.001). About half of the patients with LGBSG were treated with none/unknown adjuvant therapy (110/236, 46.6% in pediatric group and 113/204, 55.4% in adult group). And about 70% patients with HGBSG were treated with the combination of RT and CT (29/37, 78.4% in pediatric and 35/52, 67.3% in adult group). CT only were mainly applied in pediatric LGBSG group (46/49, 94%) **(** **Supplement Table 1** **).** 52.7% (n=144) of pediatric patients had pilocytic astrocytoma (PA) while 45.3% (n=116) of adult patients had ependymoma (EP). (**Supplement Table 3**). The frequency distribution between EP PA and other low grade (OLG) BSG were compare **(** **Supplement Table 4** **)**. There were several significant differences in the EP group: 68.2% (n=120) of patients received GTR; 53.4% (n=94) of patients were treated with radiotherapy only; 65.9% (n=116) of patients were adults; 46.0% (n=46.0) of patients had ventricular system involved tumor.

**Figure 2:**
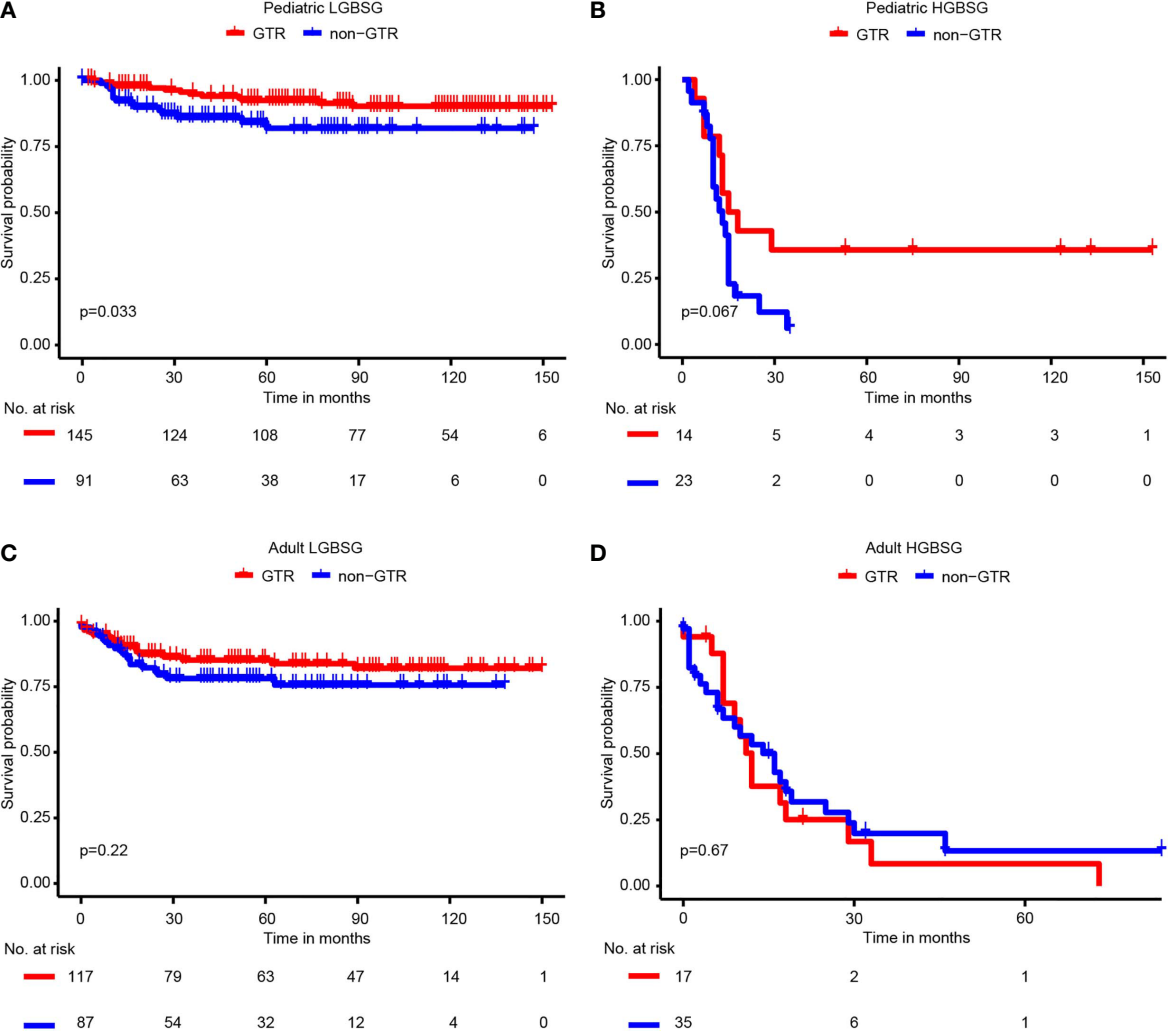
Kaplan-Meier curves by GTR and non-GTR in different patient groups. **(A)** pediatric LGBGG; **(B)** pediatric HGBSG; **(C)** adult LGBSG; **(D)** adult HGBSG patient.

Regarding to surgical resection, the Kaplan-Meier curves showed that the survival probability of GTR group was significantly higher than that of non-GTR group only in pediatric LGBSG group (P=0.033). In adult LGBSG and pediatric HGBSG group, GTR group had better survival probability compared with non-GTR group but the differences were not significant (P=0.22 and 0.067, respectively). In adult HGBSG group, the survival curve showed no difference between GTR and non-GTR group (P=0.67) **(** **Figure 2** **)** Consistently, univariate and multivariable analyses in four different patient groups revealed that GTR is an independently significant predictor for prolonged CSS in pediatric LGBSG group (HR0.29, 95%CI 0.11-0.78, P=0.015); In pediatric HGBSG group, the univariate analysis showed that GTR is significantly related to better CSS (HR0.39, 95%CI 0.16-0.99, P=0.048) but lost its significance after the adjustment of multivariable analysis (HR0.60, 95%CI 0.20-1.76, P=0.350). In adult patient groups, the association between surgical resection and CSS was not statistically significant in univariate nor multivariable analysis (**Table 2**). As both univariate and multivariable analysis showed that the CSS was not statistically significant influenced by STR when compared with biopsy only in 4 different patient groups, the extent of surgery was further categorized as GTR and non-GTR.

**Table 2:** The result of univariate and multivariable analysis of extent of resection and adjuvant therapy in four different patient groups.

Patient groups	Treatment options	Univariate analysis		Multivariable analysis	
		HR (95%CI)	P-value	HR (95%CI)	P-value
Pediatric LGBSG	Surgery				
	Biopsy	1 [Reference]		1 [Reference]	
	STR	1.01 (0.35-2.94)	0.990	0.90(0.30-2.71)	0.844
	GTR	0.44(0.18-1.08)	0.072	0.29(0.11-0.78)	0.015^†^
	Adjuvant therapy				
	None/unknown	1 [Reference]		1 [Reference]	
	Radiotherapy	15.16(3.45-66.71)	<0.001^†^	22.16(4.78-102.83)	<0.001^†^
	Chemotherapy	5.04(0.92-27.53)	0.062	5.18(0.89-30.26)	0.068
	Both	14.29(2.88-70.90)	<0.001^†^	11.66(2.28-59.78)	0.003^†^
Adult LGBSG	Surgery				
	Biopsy	1 [Reference]		1 [Reference]	
	STR	0.96(0.38-2.38)	0.92	0.97(0.38-2.45)	0.95
	GTR	0.66(0.31-1.39)	0.27	0.77(0.34-1.71)	0.51
	Adjuvant therapy				
	None/unknown	1 [Reference]		1 [Reference]	
	Radiotherapy	1.07(0.51-2.24)	0.863	1.02(0.46-2.25)	0.968
	Chemotherapy	\	\	\	\
	Both	4.50(1.94-10.44)	<0.001^†^	3.98(1.61-9.81)	0.003
Pediatric HGBSG	Surgery				
	Biopsy	1 [Reference]		1 [Reference]	
	STR	0.71(0.29-1.72)	0.446	1.32(0.44-3.97)	0.623
	GTR	0.39(0.16-0.99)	0.048^†^	0.60(0.20-1.76)	0.350
	Adjuvant therapy				
	None/unknown	1 [Reference]		1 [Reference]	
	Radiotherapy	5.78(0.58-57.14)	0.13	9.08(0.67-122.71)	0.097
	Chemotherapy	/	/	/	/
	Both	6.05(0.81-45.34)	0.08	3.69(0.42-32.24)	0.237
Adult HGBSG	Surgery				
	Biopsy	1 [Reference]		1 [Reference]	
	STR	0.46(0.20-1.07)	0.073	0.53(0.16-1.79)	0.306
	GTR	0.85(0.42-1.70)	0.638	1.01(0.41-2.49)	0.991
	Adjuvant therapy				
	None/unknown	1 [Reference]		1 [Reference]	
	Radiotherapy	0.84(0.27-2.62)	0.764	0.88(0.26-2.96)	0.837
	Chemotherapy	6.79(0.74-62.55)	0.091	7.78(0.64-94.26)	0.107
	Both	0.46(0.20-1.04)	0.062	0.35(0.11-1.09)	0.070

^†^P < 0.05, statistically significant.

**Figure 3:**
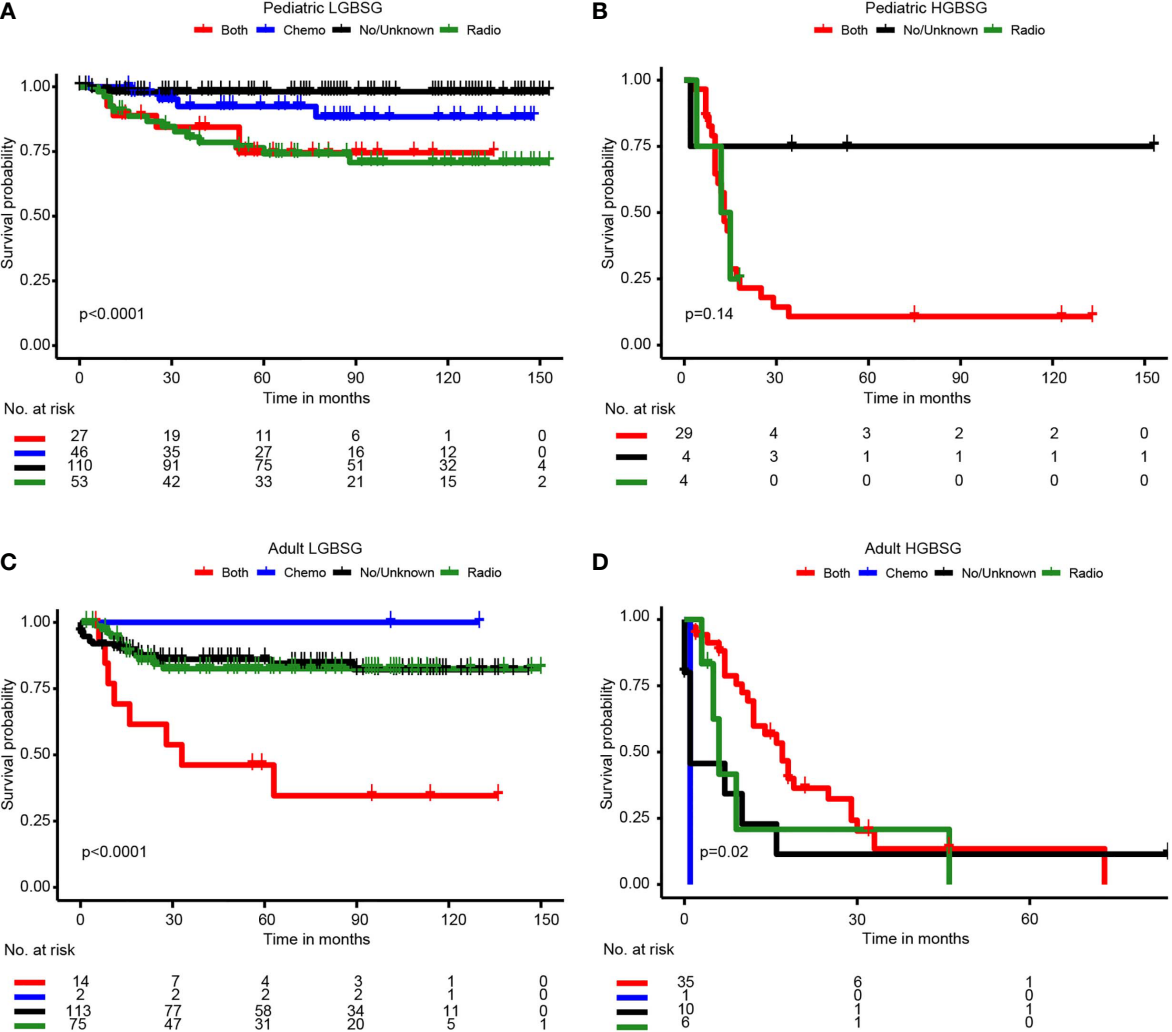
Kaplan-Meier curves by adjuvant therapy in different patient groups. **(A)** pediatric LGBGG; **(B)** pediatric HGBSG; **(C)** adult LGBSG; **(D)** adult HGBSG patient.

Concerning adjuvant therapy, Kaplan-Meier curves in different groups showed that patients treated with both RT and CT in adult HGBSG group had better survival probability. (P=0.02) And in other groups, all kinds of adjuvant therapies showed no survival advantages compared with none/unknown group (**Figure 3**). Univariate (HR0.46, 95%CI 0.20-1.04, P=0.062) and multivariable (HR0.35, 95%CI 0.11-1.09, P=0.070) analyses revealed that the combination of RT and CT was related to better CSS but not significantly in adult HGBSG group. In other patient groups all kinds of adjuvant therapies were not statistically related to better CSS or significantly related to worse CSS (**Table 2**)

## Discussion

This study has the following strengths. First, this study included 529 patients with histologically confirmed BSG, which was difficult to achieve in clinical study because of the low prevalence and histological diagnosis rate of brainstem tumor. This study highlighted the importance of histological diagnosis on the prognosis and treatment choices for BSG patients. Second, this study grouped patients based on age and WHO grade to directly compare the effects of treatment methods on prognosis separately, which provided a comprehensive understanding of BSG and important information for the choice of treatment in clinical practice. Third, this study confirmed the role of GTR in pediatric LGBSG with increased strength and low bias, which was reported by several monocentric small-scale studies but with no statistical significance.

This study also has its limitations. First, some important tumor characteristics such as the concrete position and growth pattern were not recorded in SEER database; Second, the detailed regimen of CT and dosage of RT were not recorded in SEER database; Third, As majority of patients with DIPG were not biopsied in clinical practice, our study cohort with histologically confirmed BSG only were not reprehensive for these patients. Fourth, the population size of pediatric and adult HGBSG was insufficient. Analyses of the whole HGBSG group were done and the results were presented in **Supplement Figure 2** and **Supplement Table 2**. Finally, the surgery-related mortality, morbidity and postoperative quality of life were also not recorded, so we couldn’t access the risk of the radical surgical resection of BSG. The postoperative mortality was 0 in most clinical studies and the percentage of postoperative sequelae ranged from 11-36.5% (**Supplement Table 5**).

Pediatric LGBSG group had the most favorable 5-year CSS rate of 88.6% in our cohort, which ranged from 67% to 100% in different reported pediatric LGBSG cohorts (15–17). The 5-year CSS rate of pediatric HGBSG group was 18.6% in our cohort. In a cohort with 13 pediatric patients with LGBSG and 5 patients with HGBSG, the 1-year OS was significantly worse in patients with HGBSG (90% vs 0%, p<0.0005) (24). Another study including 44 pediatric patients with intrinsic BSG reported that the 1-year OS rates of patients with LGBSG and HGBSG were 80.4% ± 0.08% and 48.6% ± 0.14%, respectively (25). For adult patients, the 5-year CSS rates of LGBSG and HGBSG group were 80.6% and 12.8% in our cohort. A cohort with 104 histologically confirmed adult patients reported the 5-year OS rates for WHO grade I, II, III and IV were 63%, 40%, 18% and 0 respectively (22). Generally, the results of clinical studies of patients with histological diagnosis were consistent with ours in that pediatric LGBSG group had the best clinical outcome.

The survival benefits of surgical resection for patients with BSG were still unproven. At the time of data collected, the CSS rates of biopsy, STR and GTR group were 63.8%, 70.6% and 81.5% respectively (P<0.001). GTR rate was significantly higher for LGBSG (P<0.001) and slightly higher in ventricular system and other involved tumors (P=0.059). EP accounted for 33.3% of cohort with the highest GTR rate (68.2%). Notably, there were 46.0% (n=81) of EP involved ventricular system, which was the highest compared with other histologic types. A Chinese cohort with 311 patients treated with surgical resection, the GTR rates for LGBSG (n=224) and HGBSG (n=86) were 47.3% and 23.3% respectively. Consistently, EP had the highest GTR rate of 76.3% in this cohort (14). Clinical studies also suggested that patients with focal low grade brainstem and dorsally exophytic tumor might benefit from surgical resection (26, 27). These results suggested that EP was more amenable to surgical resection in clinical practice. As our cohort strictly included patients with histological diagnosis and most of patients were treated with surgical resection (402/529, 75.9%), this can partially explain the high percentage of EP in our cohort. A retrospective analysis of 104 adult patients with histologically confirmed BSG reported that only 2 (1.9%) patients had EP. But there were only 11 (10.6%) patients treated with surgical resection while 93 (89.4%) patients received biopsy (22). Our analyses revealed that GTR was an independent favorable predictor for CSS only in pediatric LGBSG group. Consistent with our results, mayo clinic reported that the 5-year survival was significantly improved when undergoing resection group was compared with biopsy group (85% vs 50%, P=0.002) (17). However, most of the clinical studies of pediatric LGBSG concluded that GTR was associated with better survival which was not statistically significant. The most important reason might be the limited sample size (16, 28). A prospective cohort of 116 pediatric LGBSG patients (German SIOP-LGG 2004 cohort) reported that higher extent of resection yielded lower progression rate (P=0.0001) but wasn’t associated with better OS (21).Besides, several small monocentric series with selective pediatric LGBGS patients also reported that patients treated with GTR had improved survival (18, 29). For pediatric patients with HGBSG, our analyses revealed there was difference in survival associated with GTR but the difference was not statically significant. The limited patient number (n=37) of this group may be the major reason. And there are still no clinical studies focused on pediatric HGBSG group. Several studies including pediatric patients with mixed grade BSG reported that patients with HGBSG had worse survival than those who had LGBSG after surgical resection. However, we can’t determine the survival benefits of surgical resection for pediatric HGBSG group based on these studies (24, 25, 30). The Kaplan-Meier curves showed GTR group had better survival probability compared with non-GTR group in adult LGBSG group but not significantly (P=0.067). And in adult HGBSG group, there were no difference between GTR and non-GTR group (P=0.67). Most clinical studies for adult patients with BSG included few patients treated with surgical resection (range from 0-14%) (21, 22, 31). A cohort with 16 consecutive adult patients treated with surgical resection, 2 of which achieved GTR, concluded that the surgical resection was of questionable benefits for adult patients (32). Another cohort including 47 adult patients treated with surgical resection reported that the median survival time for patients in GTR group (n=14, 30%) and non-GTR group (n=33, 70%) was 47 and 37 months respectively (13). Our results and clinical studies suggested that GTR can provide significant survival benefit for pediatric LGBSG group. More clinical studies with histological diagnosis were needed to further investigate the role of GTR for patients with BSG.

The role of adjuvant therapy in treatment of BSG remains to be controversial. About half of patients with LGBSG were treated with none/unknown adjuvant therapy. And around 70% of patients with HGBSG were treated with both RT and CT. Notably, 72.0% (n=144) of PA were treated with none/unknown adjuvant therapy and 53.4% of (n=94) EP were treated with RT only. There was no reliable evidence showing that patients with BSG can benefit from adjuvant therapy (33). K-M analysis revealed improved CSS for patients who received both CT and RT compared to patients who received only CT, only RT or unknown/no therapy, however this was not evident in the multivariable analysis (p=0.07). The limited sample size may be the major reason. In other patient groups, adjuvant therapy was not related to survival or significantly related to worse CSS. Given the fact that adjuvant therapy was preferentially performed in patients treated with biopsy only or STR, this selection bias may be an important reason (17, 34). A clinical study including 96 pediatric patients with LGBSG reported that upfront adjuvant therapy did not significantly improve the prognosis of patients with residual tumor compared with observation only (35). The German SIOP-LGG 2004 cohort of pediatric patients with LGBSG also reported that more than half of the patients could be safely followed by observation even with high percentage of STR (90%) (15). Generally, Clinical studies of pediatric BSG concluded that adjuvant therapy did not provide survival benefits (36–38). A retrospective analysis of 104 adult histological proven adult BSG patients reported that RT (HR 0.4, 95%CI 0.2-0.9, P=0.021) and RT combined with CT (HR 0.3, 95%CI 0.1-0.9, P=0.041) were significantly related to better survival. However, this study didn’t analyze adult LGBGS (n=47) and HGBSG groups (n=57) separately (22). A large-scale population-based study including 422 adult patients with histologically confirmed HGBSG from National Cancer Database reported that RT combined with CT was significantly related to better CSS (RT and CT versus RT, HR 0.67, 95%CI0.46-0.98, P=0.038), while the RT only was not statistically related to CSS (none vs RT, HR 1.25, 95%CI0.82-1.89, P=0.30) (39). Compared with our results, this study proved that combination of RT and CT provided survival benefits in adult HGBSG group with more strength. For adult LGBSG group, more clinical studies were needed.

## Conclusion

This study is the first large-scale population-based study of BSG comparing the clinical outcomes and treatment options directly between patient groups with different age and WHO grade. Pediatric LGBSG group had the highest GTR rate and most favorable clinical outcome. GTR can provide survival benefits for pediatric LGBSG group. This study provides valuable data for further clinical study and highlights the need for more clinical studies with histological diagnosed BSG.

## Data Availability Statement

The original contributions presented in the study are included in the article/**Supplementary Material**. Further inquiries can be directed to the corresponding authors.

## Author Contributions

ZYL, QC, and SS made substantial contribution to the design of this study. ZYL and QC carried out the analysis and interpreted the data. ZYL and SS made contributions to the drafting of the manuscript. JL and HC made contributions to the review of previous literature. JH, FF, and LC contributed substantially to the revision of the manuscript. QC and ZXL made substantial contributions to the conception of the manuscript, and were responsible for the quality of the overall manuscript. All authors contributed to the article and approved the submitted version.

## Funding

This work was supported by the National Natural Science Foundation of China (NO.82073893, NO.81703622, NO. 81472693, NO. 81873635), China Postdoctoral Science Foundation (NO. 2018M633002), Hunan Provincial Natural Science Foundation of China (NO.2018SK2101, No. 2020JJ8111, NO.2018JJ3838), Hunan provincial health and Health Committee Foundation of China (C2019186).

## Conflict of Interest

The authors declare that the research was conducted in the absence of any commercial or financial relationships that could be construed as a potential conflict of interest.
