# Supplementary material for: The Survival Benefits of Surgical Resection and Adjuvant Therapy for Patients With Brainstem Glioma
Source: Front Oncol. 2021 Mar 25;11:566972. doi: 10.3389/fonc.2021.566972 (PMC8027112; doi:10.3389/fonc.2021.566972)
Supplement: Supplementary file 1 [file DataSheet_1.pdf]

**Supplement Figure 1** Flowchart of patient with histologically confirmed brainstem glioma selection

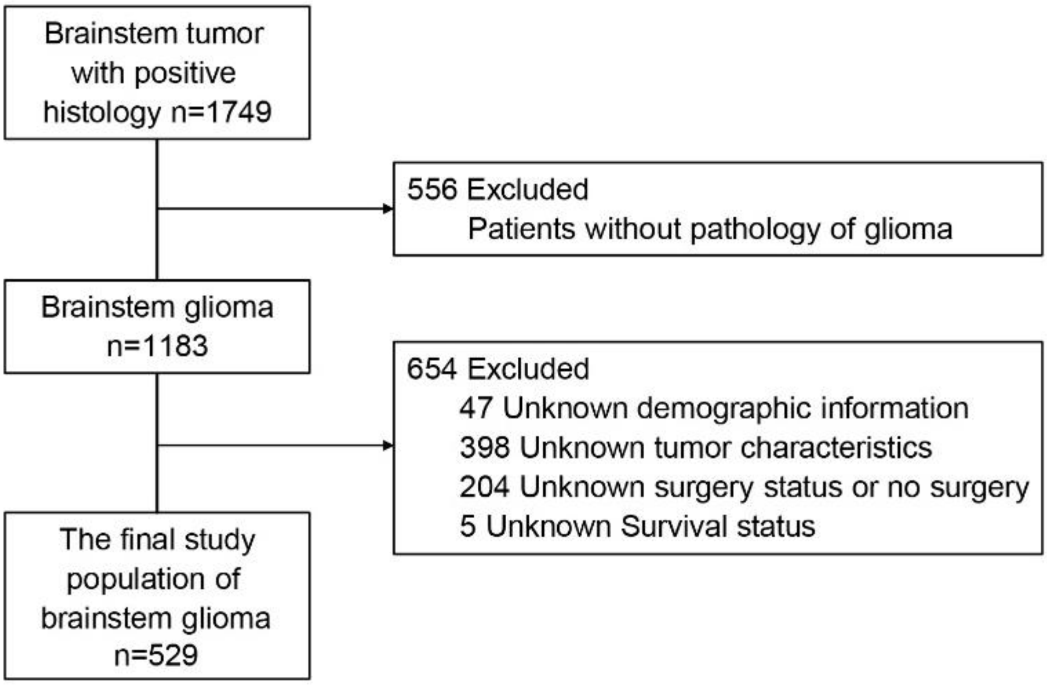

**Supplement Figure2** Kaplan-Meier curves by **(A)** adjuvant therapy **(B)** GTP and non-GTR in HGBSG patient group.

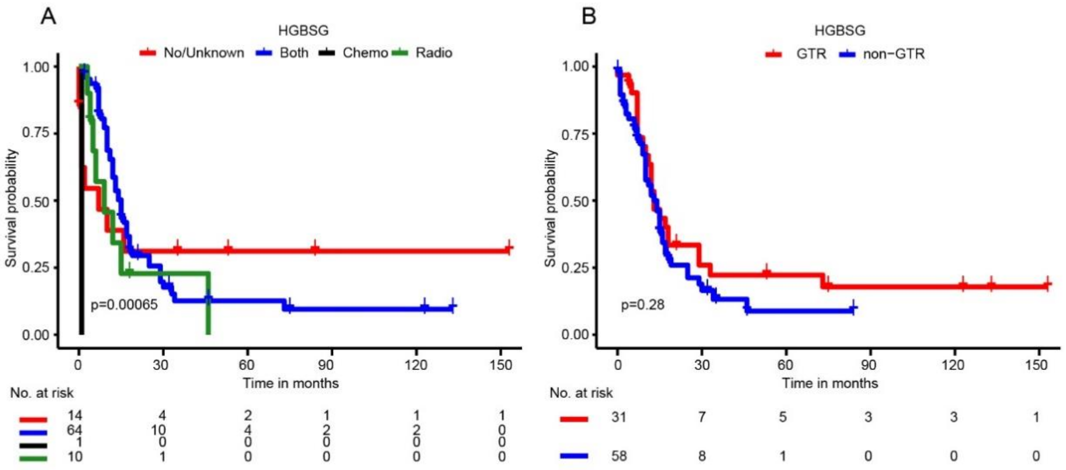

**Supplement Table 1** The distribution of extent of surgery and adjuvant therapy in 4 different patient groups

| | Pediatric LGBSG N (%) | Pediatric HGBSG N (%) | Adult LGBSG N (%) | Adult HGBSG N (%) | P-value |
|---|---|---|---|---|---|
| Population size | 236 (100) | 37 (100) | 204 (100) | 52 (100) | |
| 5-year CSS rates | 88.6% | 18.6% | 82.1% | 11.3% | |
| Extent of surgery | | | | | <0.001† |
| Biopsy | 47 (19.9) | 11 (29.7) | 48 (23.5) | 21 (40.4) | |
| STR | 44 (18.6) | 12 (32.4) | 39 (19.1) | 14 (26.9) | |
| GTR | 145 (61.4) | 14 (37.8) | 117 (57.4) | 17 (32.7) | |
| Adjuvant Therapy | | | | | <0.001† |
| No/Unknown | 110 (46.6) | 4 (10.8) | 101 (49.5) | 10 (19.2) | |
| Radiotherapy | 53 (22.5) | 4 (10.8) | 73 (35.8) | 6 (11.5) | |
| Chemotherapy | 46 (19.5) | 0 (0.0) | 2 (1.0) | 1 (1.9) | |
| Both | 27 (11.4) | 29 (78.4) | 14 (6.9) | 35 (67.3) | |

**Note:** †P<0.05, statistically significant.

**Supplement table 2** The result of univariate and multivariate analysis of extent of resection and adjuvant therapy in HGBSG patient groups

| Treatment options | Univariate analysis | | Multivariable analysis | |
|---|---|---|---|---|
| | HR(95%CI) | P-value | HR(95%CI) | P-value |
| Surgery | | | | |
| Biopsy | 1 [Reference] | | 1 [Reference] | |
| STR | 0.57 (0.31-1.03) | 0.064 | 0.67 (0.33-1.37) | 0.33 |
| GTR | 0.59 (0.33-1.02) | 0.059 | 0.74 (0.40-1.57) | 0.27 |
| Adjuvant therapy | | | | |
| None/unknown | 1 [Reference] | | 1 [Reference] | |
| Radiotherapy | 1.54 (0.58-4.02) | 0.38 | 1.29 (0.47-3.55) | 0.621 |
| Chemotherapy | 22.04(2.37-205.17) | 0.006† | 10.74(1.03-111.87) | 0.047† |
| Both | 1.02 (0.50-2.10) | 0.95 | 0.61(0.25-1.46) | 0.27 |

**Supplement Table 3** The distribution of histologic type in pediatric and adult patient groups

| | GTR rate | ALL N (%) | Pediatric N (%) | Adult N (%) |
|---|---|---|---|---|
| Population size | 55.3% | 529(100) | 273(100) | 256(100) |
| Pilocytic astrocytoma (I) | 58.0% | 200(37.8) | 144(52.7) | 56(21.9) |
| Astrocytoma, NOS (II) | 36.0% | 50(9.5) | 26(9.5) | 24 (9.4) |
| Diffuse astrocytoma (II) | 5/10 | 10(1.9) | 5(1.8) | 5(2.0) |
| Ependymoma/anaplastic ependymoma(II) | 68.2% | 176(33.3) | 60(22.0) | 116(45.3) |
| Oligodendroglioma(II) | 1/1 | 1(0.2) | 1(0.4) | 0(0.0) |
| Unique astrocytoma variants(II) | 2/3 | 3(0.6) | 0(0.0) | 3(1.2) |
| Anaplastic astrocytoma(III) | 29.6% | 27(5.1) | 11(4.0) | 16(6.3) |
| Anaplastic oligodendroglioma(III) | 1/1 | 1(0.2) | 0(0.0) | 1(0.4) |
| Mixed glioma( III) | 2/5 | 5(0.9) | 2(0.7) | 3(1.2) |
| Neuroepithelial (III) | 1/1 | 1(0.2) | 1(0.4) | 0(0.0) |
| Glioblastoma (IV) | 36.4% | 55(10.4) | 23(8.4) | 32(12.5) |

**Supplement Table 4** Patient demographics, tumor characteristics and treatment options between different histology types in LGBSG group

| **Characteristics** | EP<br>N (%) | PA<br>N (%) | OLG<br>N (%) | P-Value |
|---|---|---|---|---|
| Population Size | 176 (100) | 200 (100) | 61 (100) | |
| Age group | | | | <0.001† |
| Pediatric | 60 (34.1) | 144(72.0) | 32 (52.5) | |
| Adult | 116 (65.9) | 56 (28.0) | 29 (47.5) | |
| Sex | | | | 0.12 |
| Female | 73(41.5) | 104 (52.0) | 30 (49.2) | |
| Male | 103(58.5) | 96 (48.0) | 31 (50.8) | |
| Race | | | | 0.12 |
| Other | 10 (5.7) | 16 (8.0) | 0 (0.0) | |
| Black | 17 (9.7) | 14 (0.7) | 8 (13.1) | |
| White | 149 (84.7) | 170(85.0) | 53 (86.9) | |
| Marital status | | | | <0.001† |
| Unmarried | 96 (54.5) | 170 (85.0) | 42 (68.9) | |
| Married | 80 (45.5) | 30 (15.0) | 19 (31.1) | |
| Size | | | | 0.36 |
| <4.8cm | 128(72.7) | 157 (78.5) | 44 (72.1) | |
| ≥4.8cm | 48 (27.3) | 43 (21.5) | 17 (27.9) | |
| Extension | | | | <0.001† |
| Brainstem | 44 (25.0) | 92 (46.0) | 35 (57.4) | |
| Cerebellum | 20 (11.4) | 18 (9.0) | 6 (9.8) | |
| Ventricular | 81 (46.0) | 58(29.0) | 8(13.1) | |
| Other | 31 (17.6) | 32 (16.0) | 12 (19.7) | |
| Metastasis | | | | 0.91 |
| No | 172 (97.7) | 195 (97.5) | 59 (96.7) | |
| Yes | 4 (2.3) | 5 (2.5) | 2 (3.3) | |
| Adjuvant therapy | | | | <0.001† |
| None/unknown | 59 (33.5) | 144 (72.0) | 18 (29.5) | |
| Both | 14 (8.0) | 8 (4.0) | 18 (29.5) | |
| Radiotherapy | 94 (53.4) | 14 (7.0) | 20 (32.8) | |
| Chemotherapy | 9 (5.1) | 34 (17.0) | 5 (8.2) | |
| Surgical resection | | | | <0.001† |
| Biopsy | 31(17.6) | 41(20.5) | 22(36.1) | |
| STR | 25(14.2) | 43(21.5) | 15(24.6) | |
| GTR | 120(68.2) | 116(58.0) | 24(39.3) | |
| Vital status | | | | <0.001† |
| Alive | 143 (81.3) | 185 (92.5) | 33 (54.1) | |
| Dead | 33 (18.8) | 15 (7.5) | 32 (52.5) | |
| Cancer specific vitals | | | | <0.001† |
| Alive | 155 (88.1) | 187 (93.5) | 33 (54.1) | |
| Dead | 21 (11.9) | 13 (6.5) | 28 (45.9) | |

**Note:** $^{\dagger}P<0.05$, statistically significant.

**Supplement Table 5** Review of clinical studies of brainstem glioma with histological diagnosis

| Study | Patient group | N[a] | Surgery related Mortality /Morbidity | GTR rate | Surgical resection |
|---|---|---|---|---|---|
| Sinha S [26] | Well delineated BSG | 58 | 5%/19% | / | 90% patients improved neurological status after surgery |
| Wang CC [14] | BSG | 311 | 1.3%/27.6 % | 40.5% | LGBSG had higher GTR rate and better survival outcome after surgery |
| Teo C [19] | Focal BSG | 34 | 0/18.8% | 35.2% | LGBSG had higher GTR rate |
| Lesniak MS [32] | Contrast-enhanced Pediatric BSG | 57 | 0/22.7% | 50.8% | Juvenile pilocytic astrocytoma had the best survival outcome after surgery. |
| Mottolese C [20] | Tectal plate Pediatric BSG | 27 | 0/18.5% | 60% | Exophytic Tectal plate glioma could be treated with surgical resection safely. |
| Sun T [24] | Pediatric BSG | 33 | / | 21.2% | LGBSG has better survival outcome after surgery. |
| HolzapfelJ [15] | Pediatric LGBSG | 116 | 0/21% | 10% | GTR significantly related to better PFS but not OS. |
| Upadhyaya SA [16] | Pediatric LGBSG | 23 | 0/21.7% | 17.4% | GTR was related to better survival outcome but not significantly. |
| Sandri A [29] | Focal Pediatric LGBSG | 17 | 0/17.6% | 13.5% | GTR was significantly related to better PFS but not OS. |
| Ahmed KA [17] | Pediatric LGBSG | 48 | / | 8.3% | Surgical resection significantly improved survival outcome |
| Klimo P Jr [28] | Pediatric LGBSG | 52 | 9.6%/36.5% | 17% | GTR was significantly related to better PFS but not OS. |
| Tasic G [31] | Adult BSG | 48 | / | 3.9% | LGBSG had better survival outcome after surgery. |
| Reithmeier T [22] | Adult BSG | 104 | 0/11.6% | 2% | GTR was not significantly related to survival outcome |
| Kesari S [21] | Adult BSG | 28 | 0/29% | / | GTR was not significantly related with survival outcome |
| Majchrzak K [13] | Adult BSG | 47 | 4.2%/45% | 30% | Focal tectal and exophytic BSG and had higher GTR rate and better prognosis |

**Note:** N[a], number of patients with histological diagnosis in the study cohort.
